# Gel electrophoresis of human sperm: a simple method for evaluating sperm protein quality

**DOI:** 10.1186/s12610-018-0076-0

**Published:** 2018-09-03

**Authors:** Jumeau Fanny, Sigala Julien, Fernandez-Gomez Francisco-Jose, Eddarkaoui Sabiha, Duban-Deweer Sophie, Buée Luc, Béhal Hélène, Sergeant Nicolas, Mitchell Valérie

**Affiliations:** 1 EA 4308 - GQG – Gametogenesis and gamete quality, University of Lille, F-59000 Lille, France; 2 CHU Lille, Reproductive Biology – Spermiology – CECOS Institute, F-59000 Lille, France; 3 University of Lille, Institut National de la Santé et de la Recherche Medicale (INSERM), CHU Lille, UMR-S 1172 JPArc, F-59000 Lille, France; 4 EA 2465 - LBHE Blood-Brain Barrier Laboratory, University of Artois, F-62307 Lens, France; 5 CHU Lille, EA 2694 - Santé publique: épidémiologie et qualité des soins, University of Lille, F-59000 Lille, France; 6 Present address: Reproductive Biology Laboratory - CECOS, Rouen University Hospital, Rouen University, F-76031 Rouen, France

**Keywords:** Fibrous sheath, One-dimensional PAGE, Sperm, Electrophorèse unidimensionnelle, Gaine fibreuse, Sperme

## Abstract

**Background:**

The limitations of conventional sperm analyses have highlighted the need for additional means of evaluating sperm quality.

**Methods:**

In a study of a cohort of 245 men with known conventional sperm parameters, one-dimensional PAGE was used to monitor protein content and quality in samples from individual ejaculates.

**Results:**

The sperm protein content varied markedly from sample to another, especially in the high-molecular-weight range. The intensity of the 80–110 kDa bands was correlated with progressive motility (*r* = 0.15, *p* = 0.015*)* and was significantly higher (*p* = 0.0367) in the group of men with conventional parameters above the World Health Organization’s 2010 reference values than in the group with at least one subnormal parameter (i.e. semen volume, sperm concentration, sperm count per ejaculate, progressive motility, proportion of normal forms or multiple anomaly index below the lower reference value). Using mass spectrometry, the 80–110 kDa bands were found to correspond primarily to three proteins from the flagellum’s fibrous sheath: A-kinase anchor protein 4, A-kinase anchor protein 3, and spermatogenic cell-specific type 1 hexokinase.

**Conclusion:**

One-dimensional PAGE constitutes a simple, rapid, reliable, inexpensive method for analyzing proteins associated with sperm motility in individual human ejaculates.

## Résumé

**Contexte:**

Les limites des analyses conventionnelles du sperme ont mis en évidence la nécessité de moyens supplémentaires d’évaluation de la qualité du sperme.

**Matériel et Méthodes:**

La méthode d’électrophorèse unidimensionnelle a été utilisée pour évaluer la teneur et la qualité protéique dans des échantillons d’éjaculats individuels de 245 hommes avec des paramètres spermatiques connus.

**Résultats:**

La teneur en protéines du sperme variait d’un échantillon à l’autre, en particulier dans la gamme des poids moléculaires élevés. L’intensité des bandes de 80–110 kDa était corrélée à la mobilité progressive (*r* = 0,15, *p* = 0,015) et était significativement plus élevée (*p* = 0,0367) dans le groupe des hommes avec des paramètres conventionnels spermatiques supérieurs aux valeurs de référence (OMS, 2010) comparé au groupe d’hommes dont l’éjaculat présentait au moins un paramètre spermatique (volume de sperme, concentration de spermatozoïdes, numération des spermatozoïdes, motilité progressive, formes normales et indice d’anomalies multiples) en deça de la valeur de référence. Les bandes dans la gamme de 80–110 kDa ont été identifiées en spectrométrie de masse et se sont révélées être des protéines de la gaine fibreuse du flagelle: la protéine d’ancrage A-kinase 4, la protéine d’ancrage A-kinase 3 et l’hexokinase de type 1.

**Conclusion:**

La méthode d’électrophorèse unidimensionnelle constitue une méthode simple, rapide, fiable et peu coûteuse pour analyser la qualité des protéines du sperme dans les éjaculats humains individuels.

## Background

At present, sperm quality is primarily evaluated in terms of conventional parameters like volume, concentration, motility, and the proportion of normal forms. However, some infertility-related aspects of sperm dysfunction are not always apparent in a conventional semen analysis [1–3]. Indeed, some infertile men may have conventional sperm parameters above the reference limit quoted in the World Health Organization (WHO) guidelines [4]. Hence, the limitations of conventional sperm analyses have highlighted the need for additional means of evaluating sperm quality.

Sperm proteins are involved in many cellular processes, such morphology, motility, capacitation, the acrosomal reaction, sperm-oocyte interactions, and signal transduction. Given that very little de novo protein synthesis occurs in the spermatozoon, any differences in protein quantity or quality may reflect a change in biological function. Extensive, high-precision, proteomic analyses of human spermatozoa have identified a vast number of sperm proteins. The sperm cell proteome provides an integrated overview of the major biological pathways required for sperm function [5]. However, the need for technical skills and sophisticated, expensive equipment rules out the routine use of this technique in hospital reproductive biology labs.

The objectives of the present study were to (i) develop a PAGE-based molecular method for investigating semen quality (as an adjunct to conventional sperm analysis) and (ii) search for putative correlations between conventional semen parameters and the sperm protein pattern. Our laboratory recently developed a method for extracting human proteins from individual sperm ejaculates [6]. One-dimensional PAGE provides a single-step overview of sperm proteins. This easy-to-perform protein analysis may be of particular value for evaluating sperm quality in individual ejaculates. Hence, the objectives of the present study were to (i) use one-dimensional PAGE to assess sperm protein content in samples from a representative cohort of 245 men with known conventional sperm parameters, (ii) determine whether or not differences in the band patterns on Coomassie-blue-stained gels were correlated with conventional sperm parameters, and (iii) use mass spectrometry to identify proteins whose expression varied markedly from one individual sperm ejaculate to another.

## Methods

### Subjects

Fresh semen samples were obtained from 245 men presenting for semen evaluation at our hospital’s Assisted Reproduction Unit (Lille University Medical Center, Lille, France). All samples were evaluated with regard to (i) conventional parameters (semen volume, sperm concentration, sperm count per ejaculate, motility, and vitality), according to the WHO guidelines [4], and (ii) sperm morphology, according to David’s modified classification [7].

### Extraction of sperm proteins

Each fresh semen sample was prepared for protein analysis as described previously [6]. Seminal plasma, immature germ cells, and non-sperm cells were removed by centrifugation (350×g, for 20 min at room temperature) through 50% PureSperm (Nidacon, Mölndal, Sweden)/FertiCult medium (FertiPro N.V., Beernem, Belgium). The sperm pellet was washed once by resuspension in 1 mL of Tris-buffered saline (10 mM Tris-HCl pH 7.6, 100 mM NaCl) and further centrifugation (350×g, for 20 min at room temperature). The sperm pellet was then resuspended in 500 μL of lysis buffer containing 20 mM Tris, 2% SDS and 1% Nuclease Mix (GE Healthcare Life Sciences, Piscataway, NJ, USA). Lysates were sonicated on ice (40 Hz, 60 pulses) and centrifuged (14,000×g, for 20 min at 4 °C). The supernatants were recovered, and the pellets were discarded. The protein concentration was assayed according to Bradford’s method (BioRad, Hercules, CA, USA) by following the kit manufacturer’s instructions. Protein lysates were stored at − 80 °C prior to analysis.

### One-dimensional PAGE

Fifteen μg of extracted proteins from each prepared sperm sample were diluted in lithium dodecyl sulfate buffer (LDS 2×, Life Technologies, Carlsbad, CA, USA), according to the manufacturer’s instructions. Protein homogenates were boiled for 10 min, quickly centrifuged at 500×g (using a bench centrifuge), and loaded onto a 4–12% polyacrylamide gel (NuPage® Bis-Tris PreCast 12 wells, Life Technologies). Molecular weight markers (Novex and Magic Marks, Life Technologies) were loaded into the first well. Electrophoresis was performed at a constant voltage of 200 V for 1 h. Polyacrylamide gels were stained with Coomassie Blue reagent (0.1% Blue G250 BioRad, Hercules, CA, USA, 50% ethanol (*v*/v), 10% acetic acid (v/v) and H_2_0). The protein bands were visualized after destaining with 7% (v/v) acetic acid and 10% ethanol in H_2_O. The gels were digitized with a Perfection V750 Pro scanner (Epson France, Levallois-Perret, France) and Photoshop Element 9 software (Adobe, Paris, France). To evaluate a potential effect of the protein concentration, larger amounts of total protein (i.e. 20, 25 and 30 μg) from sperm samples were loaded onto 4–12% acrylamide gels and stained with Coomassie Blue reagent. To inhibit seminal proteases, sperm samples were treated with a protease inhibitor cocktail (cOmplete™, Roche Diagnostics GmbH, Mannheim, Germany), according to the manufacturer’s instructions. One-dimensional PAGE was performed twice on each independent sample. The densitometric intensity of the molecular weight bands between 110 and 80 kDa was quantified using ImageJ software (version 1.47c, NIH). Data were expressed in arbitrary units.

### Protein identification

Coomassie-Blue-stained protein bands were trypsinized, and nanoseparation was performed with a U3000 nano-high-performance liquid chromatography (LC) system (Dionex-LC-Packings, Sunnyvale, CA, USA). After a pre-concentration step (using a C18 cartridge, 300 μm, 1 mm), peptide samples were separated on a Pepmap C18 column (75 μm, 15 cm) by applying an acetonitrile/0.1% TFA gradient: 0% acetonitrile for 3 min, 0 to 15% for 7 min, 15 to 65% for 42 min, 65 to 90% for 5 min and, lastly, 90% acetonitrile for 6 min. The flow rate was set to 300 nL/min, and the fractions were automatically collected every 30 s (giving 110 fractions in total) on an AnchorChip™ MALDI target by using a Proteineer™ FC fraction collector (Bruker Daltonics, Bremen, Germany). Two μl of α-cyano-4-hydroxycinnamic acid matrix (0.3 mg/mL in acetone:ethanol; 0.1% TFA-acidified water, 3:6:1 *v*/v/v) were added during the collection step. Mass spectrometry (MS) and MS/MS measurements were performed off-line using an Ultraflex™ II time-of-flight (TOF)/TOF mass spectrometer (Bruker Daltonics). The spectrometer’s parameters are given in the following section. Peptide fragmentation was driven by Warp LC™ software (Bruker Daltonics), using the following parameters: signal-to-noise ratio > 15, more than three MS/MS steps per fraction if an MS signal was available, a 0.15 Da tolerance for peak merge, and the elimination of peaks that appeared in more than 35% of the fractions.

Measurements on an Ultraflex™ II TOF/TOF instrument were performed in automatic mode (using FlexControl™ 3.0 software, Bruker Daltonics) for molecular mass determination, in reflectron mode for MALDI-TOF peptide mass fingerprinting (PMF), and in “LIFT” mode for MALDI-TOF/TOF peptide fragmentation fingerprinting (PFF). External calibration over a 1000–3200 mass range was performed using [M + H] + monoisotopic ions of bradykinin 1–7, angiotensin I, angiotensin II, substance P, bombesin and adrenocorticotropic hormone (clip 1–17 and clip 18–39), using a peptide calibration standard kit (Bruker Daltonics). Briefly, MS spectra were obtained with an accelerating voltage of 25 kV, a reflector voltage of 26.3 kV, and a pulsed ion extraction of 160 ns. Each individual spectrum was produced by accumulating data from 800 laser shots. A maximum of five precursor ions per sample was chosen for LIFT-TOF/TOF MS/MS analysis. Precursor ions were accelerated to 8 kV and selected in a timed ion gate. Metastable ions generated by laser-induced decomposition were further accelerated by applying 19 kV in the LIFT cell, and their masses were measured in reflectron mode. Peak lists from MS and MS/MS spectra were generated using Flexanalysis™ 3.3 software (Bruker Daltonics). Combined PMF and PFF searches in the UnitProt 2012_06_database (ProteinScape™ 2.1, Bruker Daltonics) were performed with Mascot 2.3.02 (Matrix Science Ltd., London, UK). Taxonomic analysis was restricted to human sequences. A mass tolerance of 75 ppm and 1 missing cleavage site (for PMF) and an MS/MS tolerance of 0.5 Da and 1 missing cleavage site (for MS/MS) were allowed. Carbamidomethylation of cysteine and oxidation of methionine residues were considered to be fixed and variable modifications, respectively. The relevance of protein identities was judged according to the probability-based molecular weight search score; the threshold for statistical significance was set to *p* < 0.05.

### Statistical analysis

Statistical analysis was performed using SAS software (version 9.3, SAS Institute Inc., Cary, NC, USA). Quantitative data were presented as the median [interquartile range (IQR)]. The normality of the data distribution was checked with a Shapiro-Wilk test. Quantitative semen variables were compared by applying Student’s t test. Quantitative densitometry values for the gels were compared in a Kruskal-Wallis test. Potential associations between the densitometry values and the semen variables were analyzed using Spearman’s test. The threshold for statistical significance was set to *p* < 0.05.

## Results

### Conventional parameters

For the population as a whole, the median [IQR] (range) age (*n* = 245) was 34 [31–38] (22–58) years, the semen volume (ml) was 3.7 [3–4.7] (1.15–12), the sperm concentration (10^6^/mL) was 75.0 [50–111] (1–420), the sperm count (10^6^/ejaculate) was 283.8 [192–423] (2.80–2100), the progressive motility (PR, %) was 55 [45–60] (1–80), and the proportion of normal forms (%) was 33 [25–42] (0–68).

### One-dimensional PAGE profiling

**Fig. 1:**
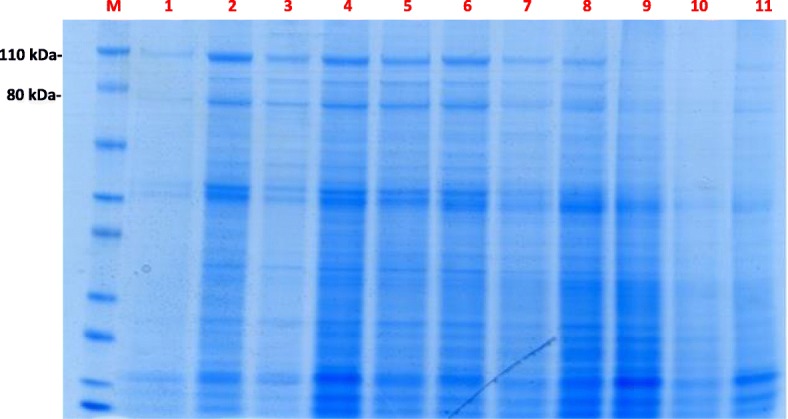
Membrane protein bands from human sperm extracts. Fifteen μg of proteins extracted from individual samples were loaded in each lane, separated on a 4–12% polyacrylamide gel, and stained with Coomassie Blue. Lanes: M: molecular mass markers; 1–11: 11 representative human sperm extracts

**Fig. 2:**
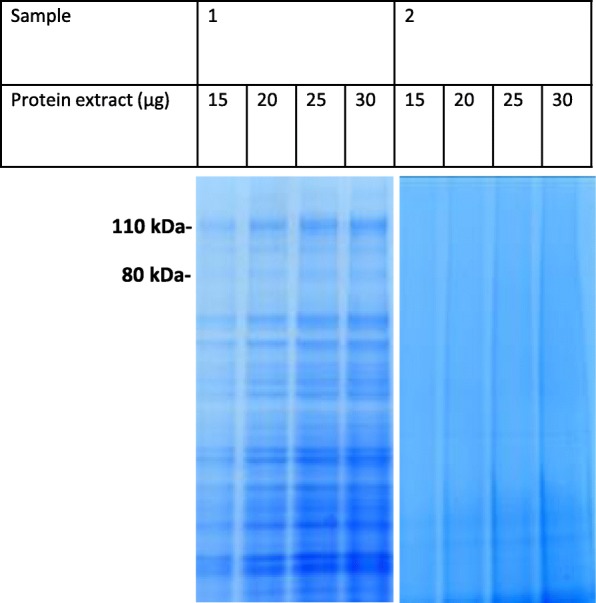
Membrane protein bands from two representative human sperm extracts (**a**, **b**). Fifteen, 20, 25 and 30 μg of proteins extracted from individual samples were loaded in each lane, separated on a 4–12% polyacrylamide gel, and stained with Coomassie Blue. **a**: stained bands at 110 and 80 kDa; **b**: absence of bands at 110 and 80 kDa

**Fig. 3:**
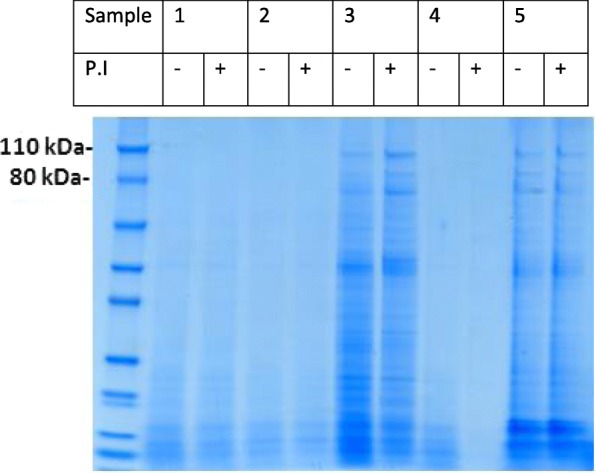
Five representative human sperm extracts (1–5) that had been treated (P.I+) or not (P.I-) with a protease inhibitor cocktail. Fifteen μg of proteins extracted from individual samples were loaded in each lane, separated on a 4–12% polyacrylamide gel, and stained with Coomassie Blue. PI: protease inhibitor

One-dimensional PAGE and Coomassie blue staining was performed for each individual semen samples. Fig. 1 shows the PAGE results for 11 samples that were representative of the population as a whole. Sperm parameters that distinguished individuals 1 to 11 of Fig. 1 were in Table 1. Sperm samples were characterized by the presence of protein bands ranging from 15 to 110 kDa. The presence and intensity of Coomassie-blue-stained bands appeared to vary markedly from one ejaculate to another. For example, the bands shown in Fig. 1 are intensely stained in lane 2, less intensely stained in lanes 1, 3 and 7, and very faintly stained in lanes 10 and 11. Increasing the sperm protein loading (20, 25 or 30 μg per lane) did not affect the band profile between 110 to 80 kDa (Fig. 2). Lastly, samples treated with a protease inhibitor cocktail gave the same patterns as non-treated samples (Fig. 3).

**Table 1:** Sperm parameters that distinguished individuals 1 to 11 of Fig. 1

Sperm parameter	Patient number (*n* = 11)											
	1	2	3	4	5	6	7	8	9	10	11	Mean ± SD
Semen volume (mL)	3.8	5.2	3	3.8	4.1	7.6	3.7	6.2	2.9	3.4	2.8	4.22 ± 1.51
Sperm concentration (×10^6^/mL)	81.5	220	210	51	52	36.5	214	58	90.5	67	300	125.5 ± 91.8
Sperm count (×10^6^/ejaculate)	309.7	114	630	193.8	213.2	277.4	791.8	359.6	262.4	227.8	840	477.2 ± 322.3
Progressive motility (PR, %)	40	60	55	55	65	80	45	40	40	60	60	54.5 ± 12.5
Normal forms (%)	28	55	57	4	21	34	31	16	28	47	54	34.1 ± 17.3
MAI	1.79	1.35	1.51	1.95	1.71	1.58	1.59	1.75	1.69	1.49	1.48	1.62 ± 0.17

*MAI* multiple anomaly index, *n* number

### Relationship between conventional sperm parameters and the protein profile obtained in one-dimensional PAGE

The densitometric intensity of the Coomassie-Blue-stained bands at 110 to 80 kDa was analyzed for all samples. In the study population as a whole (*n* = 245), the intensity of the bands at 110 and 80 kDa was correlated with the PR motility (*r* = 0.15, *p* = 0.015) but not with the sperm concentration (*r* = − 0.013, *p* = 0.834), the sperm count per ejaculate (*r* = − 0.005, *p* = 0.939) or the proportion of normal forms (*r* = 0.10, *p* = 0.116).

With a view to compare protein profiles in normozoospermic samples vs. samples with at least one subnormal sperm parameter, the study population was divided into two groups (Table 2). All the samples included in group 1 (normozoospermic, *n* = 122) had values above the WHO’s lower reference values for all parameters. All the samples included in group 2 (abnormal, *n* = 123) had a value below the WHO’s lower reference limit for one or more parameters, as follows: semen volume (*n* = 2), sperm concentration (*n* = 6), sperm count per ejaculate (*n* = 2), PR motility (*n* = 22), proportion of normal forms (*n* = 53), and multiple anomaly index (*n* = 114). The median [IQR] sperm concentration, sperm count per ejaculate, PR motility and normal forms were significantly higher in group 1 than in group 2 (Table 1). Likewise, the median [IQR] band intensities at 110 and 80 kDa were significantly higher in group 1 than in group 2 (*p* = 0.0367).

**Table 2:** Categorization of sperm samples as a function of conventional sperm parameters and a densitometric analysis of the 110–80 kDa proteins

Parameter (units)	Group 1 *n* = 122	Group 2 *n* = 123	*p*-value in Student’s t test or (when underlined) a Kruskal-Wallis test
Semen volume (mL)	3.5 [2.9–4.7] 4.07 (1.8–12)	3.8 [3.1–4.6] 4.02 (1.15–8.30)	0.81
Sperm concentration (×10^6^/mL)	78.75 [52.5–142] 106.78 (24–420)	72 [41–92] 75.99 (1–250)	0.0002
Sperm count (×10^6^/ejaculate)	319.30 [212.8–498] 405.96 (57–2100)	262.45 [155.8–356.9] 293.49 (2.80–1425)	0.0011
Progressive motility (PR, %)	55 [45–80] 54.80 (35–80)	45 [40–55] 46.28 (1–70)	< 0.0001
Normal forms (%)	40.5 [34–48] 41.61 (25–68)	25 [18–53] 23.98 (0–53)	< 0.0001
MAI	1.50 [1.44–1.55] 1.48 (1.25–1.55)	1.71 [1.64–1.80] 1.74 (1.38–1.80)	< 0.0001
80–110 kDa band intensity from gel 1D (arbitrary units)	36,298 [21996–90,482]	33,466 [16657–48,560]	0.0367

Data on semen parameters are quoted as the median [IQR] and the mean (range)

*MAI* multiple anomaly index, *n* number

### LC MS/MS protein identification

The Coomassie-blue-stained protein bands at 110 and 80 kDa were removed from the gels and analyzed using nano LC-MALDI-TOF-MS/MS. Three major proteins were identified (Table 3): spermatogenic cell-specific type 1 hexokinase (HK1S), A-kinase anchor protein 3 (AKAP3), and A-kinase anchor protein 4 (AKAP4).

**Table 3:** Proteins contained in the 110 and 80 kDa bands, following in-gel trypsinization and mass spectrometry identification

Accession	MW [kDa]	pI	Score	#Pept. [n]	SC [%]	Protein
AKAP3_HUMAN	94.7	5.8	250.2	4	5.7	A-kinase anchor protein 3
HK1_HUMAN	102.4	6.4	118.6	2	3.2	Hexokinase 1
AKAP4_HUMAN	94.4	6.6	477.1	6	9	A-kinase anchor protein 4

*MW* theoretical molecular weight, *pI*: isoelectric point, *Score* identification score, *#Pept* number of peptides sequenced, *SC* overlap with the protein sequence

## Discussion

In the present study, we analyzed the proteins in sperm samples from men with known conventional parameters. The proteins in individual (i.e. non-pooled) sperm samples were extracted, separated and visualized (using one-dimensional PAGE and Coomassie blue staining), as described previously [6]. Unexpectedly, the sperm protein content varied from one sample to another; this was especially true in the 110–80 kDa range. Furthermore, the levels of 110 and 80 kDa proteins were higher in samples with normal parameters than in samples with abnormally low parameters. Although one-dimensional PAGE has been used to identify seminal proteins [8], this method has not previously been used as a rapid analysis of sperm protein quality. It is noteworthy that the simple method used in the present study is easily applied to the investigation of protein sperm quality.

The same amount of total sperm protein was loaded into each lane of the gel lane. Nevertheless, we found that the band profile differed markedly from one individual sperm sample to another. Seminal plasma contains enzymes that cleave proteins [9]. In the present study, the sperm proteins were isolated immediately after sperm liquefaction. Nevertheless, the spermatozoa might have undergone partial molecular changes; this could explain the inter-individual changes in protein content. The inter-individual differences in the one-dimensional PAGE profile were more prominent for the relatively high-molecular-weight proteins at 110 and 80 kDa than for proteins with a lower molecular weight. However, sperm samples treated with a protease inhibitor cocktail gave much the same pattern as non-treated samples – showing that the interindividual differences in the one-dimensional PAGE profile were not due to variations in seminal protease activity.

We found that the intensities of the 80 and 110 kDa protein bands were correlated with sperm motility. In contrast, the band intensities were not related to sperm concentration, sperm count per ejaculate or the proportion of normal forms. An MS analysis of the Coomassie blue gel revealed the presence of three major proteins (AKAP4, AKAP3 and HK1S) in the 100 and 80 kDa bands. All three proteins are major components of the sperm flagellum, and AKAP4 and AKAP3 are major regulators of flagellar motion and sperm motility; this functional activity probably explains the correlation between the intensity of the 80–110 kDa bands and sperm motility. Further investigation of sperm protein expression will be required to understand the absence of a correlation between the intensity of the 80–110 kDa bands on one hand and the sperm concentration and morphology on the other. Although AKAP4 is specifically expressed in spermatozoa, it is expressed as an 854 aa (110 kDa) precursor protein. The first 188 aa are then cleaved to produce the mature (82 kDa) AKAP4. Hence, further analysis of proAKAP4/AKAP4 and AKAP3 levels will be needed to determine the relationship between protein expression and conventional sperm parameters.

The AKAP family encompasses over 50 ubiquitously expressed proteins that interact with the protein kinase A regulatory subunit dimer. AKAPs are highly abundant in the testis, where they have important roles in sperm function in general and in the regulation of motility, sperm capacitation and the acrosome reaction in particular [10]. AKAP4 (AKAP82) and AKAP3 (AKAP110) are the two most abundant proteins in the flagellum’s fibrous sheath, and are known to mediate sperm motility [11–14]. In fact, AKAP4 accounts for nearly half the protein in mouse sperm fibrous sheaths [15]. AKAP4 can bind to AKAP3 [16]; in mutant mice, loss of AKAP4 resulted in abnormally low levels of AKAP3 [12]. Furthermore, AKAP4 is known to be crucial for sperm motility [12, 16, 17]. It is therefore not surprising that AKAP4 is expressed in a variety of species, and has been highly conserved throughout evolution [18].

The hexokinases constitute a family of glycolytic enzymes with various isoforms. Glycolysis generates the ATP required for sperm motility and male fertility [19–21]. In male germ cells, glycolytic enzymes are clustered in the fibrous sheath [22, 23]. In sperm, HK1S is the predominant isoform of hexokinase. The protein is located within the principal piece of the flagellum, where it is bound to the fibrous sheath [24, 25]. Levels of HK1S in sperm and testis have been studied in the mouse [23–25].

The fact that AKAP4, AKAP3 and HK1S are fibrous sheath components indicates that the proteins are involved in sperm motility [15]. The correlation between the intensity of the 110 and 80 kDa bands and PR motility also suggests that these high-molecular-weight sperm proteins have a role in sperm motility. Several studies have demonstrated that the fibrous sheath serves as a scaffold for signaling cascades (mediated by tyrosine phosphorylation) and glycolytic enzymes [12, 26]. Along with HK1S, glycolytic enzymes associated with the fibrous sheath include glyceraldehyde 3-phosphate dehydrogenase, aldolase 1, and pyruvate kinase [27]. The fibrous sheath is involved not only in the maintenance of flagellar elasticity but also in the regulation of flagellar motility [28, 29]. Nevertheless, the mechanism by which AKAP4, AKAP3 and HK1S regulate and coordinate sperm functions is not well understood. In order to compare the protein content and profile with conventional sperm parameters, we focused on men with well-characterized sperm samples. Our results indicate that spermatozoa from individuals with at least one conventional sperm parameter below the WHO’s lower reference limit display greater variations in protein expression than spermatozoa from individuals with normal parameters.

## Conclusion

The results of the present pilot study demonstrated that individual sperm ejaculates differ in their protein composition, as measured using a simple, one-dimensional PAGE technique. These differences were especially marked for three proteins in the 80–110 kDa range, which were identified as AKAP4, AKAP3 and HK1S. The latter proteins’ roles in important functional pathways (such as flagellum motion, capacitation, and the acrosome reaction) remain to be characterized in detail.

## Abbreviations

AKAP: A-kinase anchor protein
HK1S: Spermatogenic cell-specific type 1 hexokinase
IQR: Interquartile range
LC: Liquid chromatography
MAI: Multiple anomaly index
MS: Mass spectrometry
PAGE: Polyacrylamide gel electrophoresis
PR: Progressive motility
WHO: World Health Organisation
