# Correction to: Gel electrophoresis of human sperm: a simple method for evaluating sperm protein quality

**DOI:** 10.1186/s12610-020-00102-8

**Published:** 2020-02-13

**Authors:** Fanny Jumeau, Julien Sigala, Francisco-Jose Fernandez-Gomez, Sabiha Eddarkaoui, Sophie Duban-Deweer, Luc Buée, Hélène Béhal, Nicolas Sergeant, Valérie Mitchell

**Affiliations:** 1 EA 4308 - GQG – Gametogenesis and gamete quality, University of Lille, F-59000 Lille, France; 2 CHU Lille, Reproductive Biology – Spermiology – CECOS Institute, F-59000 Lille, France; 3 University of Lille, Institut National de la Santé et de la Recherche Medicale (INSERM), CHU Lille, UMR-S 1172 JPArc, F-59000 Lille, France; 4 EA 2465 - LBHE Blood-Brain Barrier Laboratory, University of Artois, F-62307 Lens, France; 5 CHU Lille, EA 2694 - Santé publique: épidémiologie et qualité des soins, University of Lille, F-59000 Lille, France; 6 Present address: Reproductive Biology Laboratory – CECOS, Rouen University Hospital, Rouen University, F-76031 Rouen, France

**Correction to: Basic Clin Androl**

**https://doi.org/10.1186/s12610-018-0076-0**

Following publication of the original article [1], the authors reported that the author given names and family names had been transposed. The author names in this correction article are presented correctly.

Fanny (Given name) Jumeau (Family name)

Julien (Given name) Sigala (Family name)

Francisco-Jose (Given name) Fernandez-Gomez (Family name)

Sabiha (Given name) Eddarkaoui (Family name)

Sophie (Given name) Duban-Deweer (Family name)

Luc (Given name) Buée (Family name)

Hélène (Given name) Béhal (Family name)

Nicolas (Given name) Sergeant (Family name)

Valérie (Given name) Mitchell (Family name)

## References

[1] Jumeau F, Sigala J, Fernandez-Gomez F-J (2018). Gel electrophoresis of human sperm: a simple method for evaluating sperm protein quality. Basic Clin Androl.

